# Supplementary material for: PDCSA: A parallel discrete crow search algorithm for influence maximization in social networks
Source: PLoS One. 2025 Aug 5;20(8):e0329350. doi: 10.1371/journal.pone.0329350 (PMC12324674; doi:10.1371/journal.pone.0329350)
Supplement: Attachment — Submitted filename: Response-to-Reviewers.docx [file pone.0329350.s002.docx]

**Subject**：Response to Reviewers

**Manuscript ID**: PONE-D-24-54198

**Article Title**: PDCSA: A parallel discrete crow search algorithm for influence maximization in social networks

Dear Editor,

Thank you very much for allowing us the opportunity to resubmit our manuscript and address the reviewers’ valuable comments. We have carefully revised the paper in light of all the feedback and criticisms provided by reviewers. We sincerely appreciate the Editor’s Reviewers’ professional efforts and dedication. We hope that the revisions we have made will meet with your approval.

In the revised manuscript, we have not only incorporated the reviewers’ suggestions but also thoroughly addressed each comment. In the marked-up copy of the revised manuscript:

- The text marked with **single underlines** represents revisions aimed at improving the accuracy of expression, based on the reviewers’ feedback, as well as general improvements to clarity and phrasing made during the revision process.
- The text marked with **double underlines** indicates additions made in response to the reviewers’ suggestions and comments.

Additionally, due to the addition of comparative experiments, Figs 7-9 have also been redrawn. Detailed explanations regarding how each reviewer’s comment has been addressed are provided in the point-by-point response below.

Thank you again for your consideration.

Best regards,

Lihong Han

Email: hanlh16@lzu.edu.cn

School of Statistics, Lanzhou University of Finance and Economics,

Lanzhou, Gansu, China

***Reviewer #1***

**Concern # 1:**

- The evaluation of the proposed algorithm relies solely on LIE, an objective function introduced by Gong et al. (2016) in Information Sciences (“Influence Maximization in Social Networks Based on Discrete Particle Swarm Optimization”). In other words, the paper uses a performance measure designed from a swarm intelligence optimization algorithm, to show the quality of another swarm intelligence optimization algorithm. This raises concerns about the neutrality of the evaluation.
Please include at least two additional evaluation criteria to provide a more comprehensive assessment of the algorithm’s performance. This will help ensure that the method performs well under diverse metrics and not only under those favorable to its design.

**Author response:** We feel great thanks for professional review work on our article. In this paper, the LIE (Local Influence Estimation) function [1], which is based on the three-hop theory, is used to approximate the local influence of individual nodes or set of nodes. Our work builds upon this local influence measure to perform optimization and identify the set of nodes that maximizes influence across the entire network. In response to the above issue, we provide the following clarification from three aspects.

(1) **Rationale for selecting the LIE function to evaluate node local influence.**
The theoretical foundation of the LIE evaluation function consists of two main aspects. First is the **three-hop theory**, which suggests that in social networks, an individual’s influence is largely confined within three degrees of separation (i.e., friends of friends of friends). Under the Independent Cascade (IC) propagation model, it is further assumed that the probability of influence propagation diminishes significantly beyond a three-hop distance [2]. The second foundation stems from the work of Pei et al., who found that the global influence of a single node in a network is primarily determined by its local influence within a two-hop neighborhood [3].

In the proposed PDCSA algorithm, the LIE evaluation function is employed during the iteration process to identify nodes with maximal local influence. Building on this, the discrete Crow Search Algorithm (CSA) is then utilized to mitigate the overlapping influence effect, thereby achieving the goal of identifying a globally optimal set of seed nodes for influence maximization. Therefore, in the experimental section of the paper, two comparative experiments were conducted. The first experiment involves comparing LIE values. The algorithms selected for this comparison must use the LIE value for assessing the local influence of nodes. The second experiment compares the optimal seed node sets identified by various algorithms through simulated propagation. This comparison includes a diverse range of algorithms, all evaluated under the Independent Cascade (IC) propagation model using Monte Carlo simulations. The aim is to compare the influence spread of the identified seed node sets across the entire experimental network.

(2) **Regarding the suggestion to include additional evaluation criteria.**
Since LIE is used only to assess the local influence of nodes or node sets, the main contribution of this paper lies in the optimization method for identifying the globally optimal seed set that maximizes influence spread. In the revised manuscript, we have incorporated a recently proposed algorithm called **AMPDE** [4], which also uses LIE values for local influence evaluation. We have incorporated the recently proposed AMPDE algorithm, which is based on LIE values for local influence evaluation, and have added a comparative analysis with this algorithm in the LIE value comparison experiment. Furthermore, in the influence maximization evaluation, we have included two recent algorithms — **CLDE** [5] and **AMPDE** — for comparison with the proposed PDCSA algorithm, in terms of their global influence spread performance.

(3) **Local Influence Evaluation Methods.**
Various methods have been developed to evaluate the local influence of nodes or node sets. One approach is the **Expected Diffusion Value (EDV)** [6], which assesses influence based on a node’s direct (one-hop) neighbors. Another approach is the **Local Influence Estimator (LIE)** and the **Expected Diffusion Impact Value (EDIV)** [7], both of which extend the evaluation to a two-hop neighborhood. The **EDIV** method introduces different weight coefficients for one-hop and two-hop neighbors during the calculation of local influence. These coefficients must be experimentally determined for each type of network to ensure optimal performance.

Currently, the primary methods for assessing node local influence are those that evaluate influence within a three-hop range. The variations among different local influence evaluation methods, particularly in assessing individual node influence, and the impact of various optimization strategies on the identification of globally optimal seed sets, remain areas that warrant further exploration. We are currently working on addressing these aspects in our ongoing research.

1. M. Gong, J. Yan, B. Shen, L. Ma, and Q. Cai. Influence maximization in social networks based on discrete particle swarm optimization. Information Sciences, vol. 367, pp. 600–614, 2016.

2. N. A. Christakis, J. H. Fowler. Connected: The surprising power of our social networks and how they shape our lives, Little, Brown, 2009.

3. S. Pei, L. Muchnik, J. S. Andrade Jr, Z. Zheng, H. A. Makse. Searching for superspreaders of information in real-world social media, Scientific reports. 4 ,5547, 2014.

4. Jianxin Tang, Qian DU. [An adaptive differential evolution algorithm driven by multiple probabilistic mutation strategies for influence maximization in social networks](https://www.worldscientific.com/doi/full/10.1142/S0129183124502322). International Journal of Modern Physics C, 36(6), 2025.

5. Chai, B., Zhang, R., Li, X. et al. CLDE: a competitive learning-driven differential evolution optimization for the influence maximization problem in social networks. J Supercomput. 2025; 81, 700.

6. Q. Jiang, G. Song, G. Cong, Y. Wang, W. Si, and K. Xie. Simulated annealing based influence maximization in social networks. In Proceedings of the twenty-fifth AAAI conference on artificial intelligence, 2011, pp. 127–132.

7. Chai, B., Zhang, R., Li, X. et al. CLDE: a competitive learning-driven differential evolution optimization for the influence maximization problem in social networks. J Supercomput. 2025; 81, 700.

**Concern # 2:**

- The paper compares the proposed method to four other state-of-the-art algorithms, which is commendable. However, the most recent benchmark cited dates back to 2018, with the earliest from 2003. To better demonstrate the value and potential of your approach, I recommend including comparisons with more recent methods from the past few years.

**Author response:** Thank you very much for your suggestions. We have added experiments on two latest algorithms (AMPDE, CLDE). The experimental results have been included in the revised manuscript, and the comparative figures 7-9 have also been redrawn.

**Concern # 3:**

- The manuscript would benefit greatly from language editing. Many sentences are ambiguous or difficult to understand. Hiring a professional copy editor is strongly recommended.

**Author response:** In the course of revising the manuscript, we have carefully reviewed and improved the language expression throughout the paper, with particular attention to grammatical accuracy and clarity of presentation. These linguistic improvements are marked by double-underlined text in the revised version.

**Concern # 4:**

- Please define key concepts clearly when they are first introduced. For example, the term “overlapping impact of node influence” on page 11 needs further explanation for clarity.

**Author response:** Thank you very much for your suggestion. In the revised manuscript, we have defined overlapping impact of node influence as **overlapping influence**, with detailed clarification provided in Definition 6. This revision aims to make the concept of overlapping influence more precise and clearly understood. We have also updated the relevant descriptions throughout the paper accordingly.

**Concern # 5:**

- On page 25, you state that the computational complexity of PDCSA is O(K ∙ ∙ ( ̅ + )). How does this compare with the computational complexity of the other benchmark algorithms? Including this comparison would provide helpful context for readers.

**Author response:** In the revised manuscript, we have included a comparative analysis of computational complexity based on the advanced algorithms presented in Table 1. Relevant discussion and comparison have been added to the **"Computational Complexity"** subsection to provide a more comprehensive evaluation of the proposed method.

***Reviewer #2***

**Concern # 1:**

- The section concerning the theoretical analysis of the algorithm lacks depth, particularly in analytically explaining the factors that affect optimization performance. A more detailed mathematical analysis would be beneficial (pp. 22–23).
**Author response:** Thank you for your suggestion. We have included the relevant mathematical expressions in the subsections "**Local Search Strategy**" and "**Global Exploration Strategy**" in the revised manuscript.

.

**Concern # 2:**

- Although the references to the swarm intelligence literature are sufficient, the lack of any mention of GNN-based (Graph Neural Network) methods is a significant omission. GNNs have also proven to be effective in solving influence maximization problems in large-scale networks.

**Author response:** We sincerely thank you for your insightful comment. In the previous version of the manuscript, the discussion on the application of Graph Neural Networks (GNNs) to influence maximization was indeed insufficient. To address this, we have added a new subsection titled **"Deep Learning-Driven IM Algorithms"** in the **Related Work** section, which reviews and incorporates recent advances in deep learning-based approaches to the Influence Maximization (IM) problem.

**Concern # 3:**

- There are several English language issues throughout the manuscript. Some sentences reflect translation artifacts and should be revised:
●Sentences such as “The core objective... is to locate a set of nodes of size K.” (p. 16) should be rephrased in a more academic manner.
●Phrases like “From the existing research, many investigations... have been conducted.” (p. 12) are too general and need to be made more specific and original.

**Author response:** We have carefully addressed the issues related to language expression and content accuracy as mentioned above. In addition, we have conducted a comprehensive review of similar linguistic expressions and accuracy-related aspects throughout the entire manuscript. The specific changes are marked and highlighted in the tracked-changes version of the revised manuscript.

**Concern # 4:**

- While experimental results are presented through figures (Figures 7–9), there is a lack of comparative tables displaying numerical data. Tables showing LIE values, runtime, and spread performance (e.g., “Table 3: LIE Value vs Time vs Seed Set Size”) should be included for greater clarity.

**Author response:** We sincerely thank you for your insightful suggestion. We have revised the manuscript by adding a performance summary table in the **"Conclusions"** section. This table presents key results across three main evaluation criteria, along with a brief comparative analysis to enhance the clarity of our findings.

**Concern # 5:**

- No link to the source code of the PDCSA algorithm is provided. The code (e.g., on GitHub) should be made publicly available, and this information should be clearly stated in the “Data Availability” section (pp. 6–7).

**Author response:** Based on your valuable suggestions, we will make the code of the algorithm proposed in this article publicly available on GitHub. Currently, we are in the process of registering the account and correcting and supplementing the necessary comments in the code. Once these tasks are completed, the access URL will be included in the final version of the manuscript.
